# Transforming 3D MRI to 2D Feature Maps Using Pre-Trained Models for Diagnosis of Attention Deficit Hyperactivity Disorder

**DOI:** 10.3390/tomography11050056

**Published:** 2025-05-13

**Authors:** Elahe Hosseini, Seyyed Ali Hosseini, Stijn Servaes, Brandon Hall, Pedro Rosa-Neto, Ali-Reza Moradi, Ajay Kumar, Mir Mohsen Pedram, Sanjeev Chawla

**Affiliations:** 1 Department of Electrical and Computer Engineering, Kharazmi University, Tehran 15719-14911, Iran; 2 Translational Neuroimaging Laboratory, The McGill University Research Centre for Studies in Aging, Douglas Hospital, McGill University, Montréal, QC H4H 1R3, Canada; seyyed.ali.hosseini@mail.mcgill.ca (S.A.H.); pedro.rosa@mcgill.ca (P.R.-N.); 3 Department of Neurology and Neurosurgery, Faculty of Medicine, McGill University, Montréal, QC H3A 2B4, Canada; 4 Department of Clinical Psychology, Kharazmi University, Tehran 15719-14911, Iran; 5 Department of Radiology, Perelman School of Medicine at the University of Pennsylvania, Philadelphia, PA 19104, USA

**Keywords:** ADHD diagnosis, 3D MRI to 2D feature maps, deep learning, VGG16 model, neuroimaging, convolutional neural networks

## Abstract

**Background:** According to the World Health Organization (WHO), approximately 5% of children and 2.5% of adults suffer from attention deficit hyperactivity disorder (ADHD). This disorder can have significant negative consequences on people’s lives, particularly children. In recent years, methods based on artificial intelligence and neuroimaging techniques, such as MRI, have made significant progress, paving the way for development of more reliable diagnostic tools. In this proof of concept study, our aim was to investigate the potential utility of neuroimaging data and clinical information in combination with a deep learning-based analytical approach, more precisely, a novel feature extraction technique for the diagnosis of ADHD with high accuracy. **Methods:** Leveraging the ADHD200 dataset, which encompasses demographic information and anatomical MRI scans collected from a diverse ADHD population, our study focused on developing modern deep learning-based diagnostic models. The data preprocessing employed a pre-trained Visual Geometry Group16 (VGG16) network to extract two-dimensional (2D) feature maps from three-dimensional (3D) anatomical MRI data to reduce computational complexity and enhance diagnostic power. The inclusion of personal attributes, such as age, gender, intelligence quotient, and handedness, strengthens the diagnostic models. Four deep-learning architectures—convolutional neural network 2D (CNN2D), CNN1D, long short-term memory (LSTM), and gated recurrent units (GRU)—were employed for analysis of the MRI data, with and without the inclusion of clinical characteristics. **Results:** A 10-fold cross-validation test revealed that the LSTM model, which incorporated both MRI data and personal attributes, had the best diagnostic performance among all tested models in the diagnosis of ADHD with an accuracy of 0.86 and area under the receiver operating characteristic (ROC) curve (AUC) score of 0.90. **Conclusions:** Our findings demonstrate that the proposed approach of extracting 2D features from 3D MRI images and integrating these features with clinical characteristics may be useful in the diagnosis of ADHD with high accuracy.

## 1. Introduction

Attention deficit hyperactivity disorder (ADHD) is a neurodevelopmental condition that manifests as defocusing, restlessness, and excessive reaction [1]. Since ADHD is a major public health problem among children as well as adults across the globe, it is essential to make the correct and early diagnosis [2]. However, the diagnosis and understanding of ADHD has been historically poor due to its variable manifestations and considerable overlap of its symptoms with other neurological conditions [3]. The Diagnostic and Statistical Manual of Mental Disorders-5 (DSM-5) and other diagnostic instruments such as International Statistical Classification of Diseases (ICD) and Related Health Problems rely on subjective evaluations of behavior to make a diagnosis of ADHD [4]. On the other hand, recent advancements in neuroimaging techniques have enabled objective assessments of ADHD [5].

Magnetic resonance imaging (MRI) is the mainstay for diagnosing and evaluating treatment responses in several neurological disorders [6]. Recent MRI developments have significantly improved brain imaging, offering greater detail and resolution to visualize brain structures and tissues. Combining artificial intelligence (AI), particularly machine/deep learning, with MRI images allows the development of clinical decision-making tools for more reliable and accurate disease detection, monitoring, and treatment response assessment [7,8,9]. However, model performance for ADHD diagnosis has been limited to date [10]. In a recent study, the integration of imaging features extracted from structural, functional MRIs and diffusion tensor images (DTI) achieved an area under the receiver operating characteristic (ROC) curve (AUC) of 0.69 and 0.64, respectively, for the diagnosis of early adolescent ADHD [11]. Using ADHD-200 holdout data in a study, Sen et al. [12] achieved an accuracy of 0.67. In a related study, Choi et al. [13] achieved an AUC of 0.86 in predicting ADHD.

Furthermore, there are considerable variations in the diagnostic performances of AI-based models in diagnosing ADHD compared to other neurological conditions [10], suggesting an unmet need for the development of an innovative approach to boost the model performance. In addition MRI data, it is hypothesized that the incorporation of clinical characteristics, such as, age, gender, handedness, and intellectual quotient, may further improve the diagnostic performance of CDS tools for the diagnosis of ADHD [14].

The ADHD-200 dataset (a publicly available dataset), originating from a collaborative effort coordinated by the Neuro Bureau, constitutes a pivotal resource for investigating ADHD through advanced data processing and machine learning methods. This dataset, collected from multiple international research centers, including Peking University, Bradley Hospital (Brown University), Kennedy Krieger Institute, The Donders Institute, New York University Child Study Center, Oregon Health and Science University, University of Pittsburgh, and Washington University in St. Louis, provides a comprehensive repository of MRI scans and demographic information, facilitating a nuanced exploration of ADHD. Originating as part of the ADHD-200 Consortium in 2011, this dataset encompasses clinical and neuroimaging data from diverse sources, with resting-state functional and structural MRI images from 973 patients.

An important criterion is the pre-processing of the input data. Both demographic data and MRI are crucial to guarantee the quality and integrity of the analyses. The dataset becomes more useful for deep learning purposes by way of quality control measures. This process mitigates the effects of noise and errors, thus ensuring that the derived models are as valid and generalizable as possible. Although 3D convolutional neural networks (CNNs) are implemented to work with volumetric data, they consume much more computational resources and memory [15]. Research has shown that transformation of three-dimensional neuroimaging data into two-dimensional (2D) slices is possible, which maintains critical pieces of clinical data, is computationally efficient, and requires less memory storage [16]. Furthermore, other neurodevelopmental and neurodegenerative disorders have been diagnosed using 2D models by generating the slices from the regions of interest [17]. In light of the multifaceted and distributed nature of ADHD, 2D slices retain the morphological characteristics relevant for its identification [18]. This approach makes it possible to employ already pretrained models such as the Visual Geometry Group16 (VGG16), which can effectively handle 2D images and reduce the workload [19].

In the current study, a pre-trained VGG16 network was used to extract features from MRI data. This approach allows deep learning models to utilize these extracted features, significantly reducing the computational costs of training complex, large MRI datasets. Converting 3D MRIs to 2D feature maps decreases computational cost and enables the utilization of existing 2D image processing methods, better scalability, and advanced model development. In the current study, various deep learning architectures, including CNNs, long short-term memory networks (LSTMs), and gated recurrent units (GRUs) were employed to investigate the model effectiveness from different standpoints. Consequently, the models were generated with and without inclusion of the personal attributes to test the additive value of personal attributes to diagnostic performances of these models. To ensure the predictive power and the models’ capabilities, a strict training and evaluation protocol was implemented. Additionally, 10-fold cross-validation tests were employed to assess the robustness of the findings.

The overall goal of this study is to develop a reliable, faster, and accurate AI-driven clinical decision-making tool using readily and widely available clinical and MRI data for the diagnosis of ADHD. To the best of our knowledge, this is the first study to apply a 3D-to-2D feature transformation using a pre-trained VGG16 model for ADHD diagnosis. Our approach uniquely combines anatomical MRI features with clinical variables (age, gender, IQ, handedness, comorbidities), enabling efficient classification with reduced computational cost. We hypothesized that our proposed pipeline would achieve superior diagnostic performance compared to previously developed models, making it more practical for wider clinical use.

## 2. Materials and Methods

### 2.1. Data Sources

- ADHD-200 Dataset

Before participating in the study, all participants (or their legal guardians) provided signed written consent after obtaining approval from the relevant research ethics review boards in St. Louis. These participants had no history of neurological or medical illness other than ADHD. The ADHD-200 Consortium made available a large training dataset (776 participants), collected from multiple institutions. During data collection, factors such as IQ, the presence of secondary conditions, and other demographic variables were taken into account, as they are known to influence the prevalence and characteristics of ADHD. This dataset covers individuals with typical development and those diagnosed with ADHD across three subtypes: inattentive (ADHD-I), hyperactive–impulsive (ADHD-HI), and a combination of both (ADHD-C). The diverse origins and extensive nature of the dataset offer both challenges and opportunities for developing robust diagnostic models.

The diagnostic criteria for ADHD can be found in the overview of the ADHD dataset (link: https://fcon_1000.projects.nitrc.org/indi/adhd200/index.html) (accessed on 11 October 2023). The variability in this dataset arises due to differences in using different scanners and variations in the acquisition parameters across sites. We believe that its diverse dataset makes it a valuable resource for developing more generalizable models.

To preprocess the resting-state MRI data, the Athena pipeline (link: https://neurobureau.projects.nitrc.org/ADHD200/Data.html) (accessed on 11 October 2023) was utilized. For an understanding of this pipeline’s methodology, refer to the work of Bellec et al. [20]. The resting-state MRI information in the ADHD-200 dataset also included data about each subject, such as age, gender, dominant hand preference, and intelligence scores. In this study, we considered all 592 subjects who had the availability of resting-state MRI scans and complete information about their clinical characteristics.

- Demographic Information

The ADHD-200 dataset includes a range of characteristics, such as age, gender, IQ scores, handedness, and secondary illnesses, for quality control and ADHD labeling.

Inclusion of demographic data such as age and gender in the analysis is important, as these parameters have been shown to be associated with ADHD and may eventually impact the clinical outcomes. Additionally, their integration in the final data analysis will be helpful in understanding their influence on the final outcome.

The other notable dimension for the dataset includes IQ. Earlier research has demonstrated some relationship between a lower IQ score and increased chances of ADHD manifestation [21]. Therefore, we included IQ scores as an independent variable in our model.

Interestingly, some prior research indicates that handedness may be a potential risk factor for the development of ADHD [3]. Therefore, the addition of handedness in our analysis may be a useful variable for further investigation [22].

The dataset adopts a labeling scheme for the diagnosis of ADHD, with “0” signifying typically developing children (TDC)/non-ADHD, ‘1’ referring to combined ADHD, “2” being hyperactive, and “3” depicting inattentive ADHD. Diagnostic labeling follows well-known classification, providing an opportunity to identify subtypes of ADHD in the dataset.

Although our study is sensitive to subtypes of ADHD, our primary focus was to explore ADHD at a group level. By taking a broad view, we will be able to look at the different facets of ADHD that interplay with such demographic characteristics such as age, gender, and ethnicity, which could improve the validity and applicability of our developed model.

### 2.2. Data Preprocessing

- Demographic Data Preprocessing

The ADHD-200 dataset includes two columns devoted to quality control of demographic variables, one for each data collection site. These quality control (QC) columns can take on the following values: “0” indicating “Questionable” data and “1” indicating data that “Passes” quality control. We excluded entries where both QC_Athena and QC_NIAK were equal to “0”, ensuring that only high-quality data were retained.

Another form of filtering was employed to ensure the accuracy of alignment of the label values. The labels, which represent diagnostic categories, were required to contain only legitimate numbers ranging from 0 to 3. The columns associated with handedness, age, gender, and full 4 IQ then underwent scrutiny, and any entries with unacceptable values were filtered out. Finally, a new column named LD_OR_ODD, having a value of “1” if any learning disorder (LD) or oppositional defiant disorder (ODD) appeared among the Secondary_DX, was added to the dataset. Subsequently, the labels were converted into binary classification. As the aim of our study was only to identify ADHD, without considering subtypes; we categorized anything that appeared as an abnormal case with code “1” and normal cases with code “0”.

In the next step, we extracted age, gender, handedness, full 4 IQ, and LD_or_ODD from the initial file to serve as demographic attributes.

It was converted into a range from 0 to 1 in the last step of preprocessing in preparation for model training phases.

**Figure 1:**
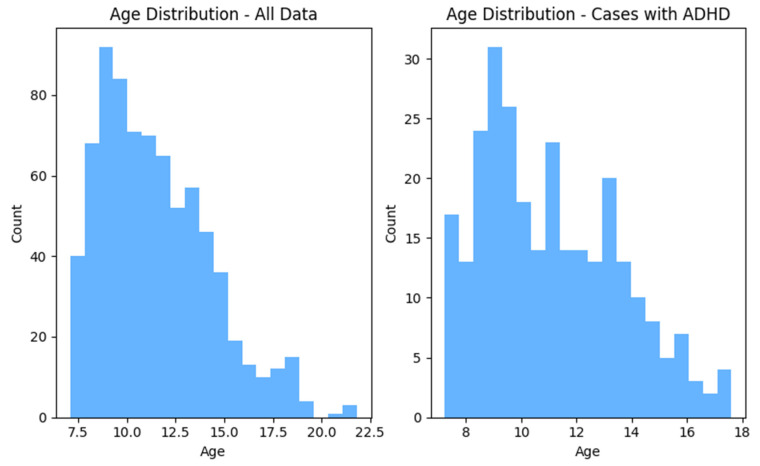
The age distribution within the whole dataset and in ADHD cases.

**Figure 2:**
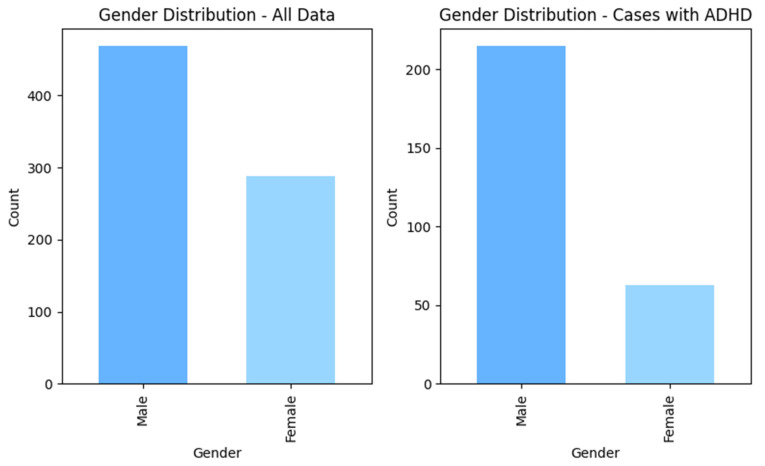
The gender distribution within the whole dataset and in ADHD cases.

After following these steps, the total number of data points was 758, including 479 non-ADHD and 279 ADHD subjects. Figure 1 and Figure 2 show the distributions of gender and age for the whole dataset and for ADHD population, respectively.

- MRI Data Preprocessing

In this study, data collected from 6 distinct sources including WASHU, OHSU, NYU, Neuroimage, KKI, and Beijing centers were used. Only T1-MPREAGE images were included from the database of each center for data analysis. Native MRI T1 images are valuable for deep learning in medical imaging due to their superior tissue contrast, high spatial resolution, and detailed anatomical structural features.

The initial step of preprocessing the MRI data included reshaping the images into 200 slices for each dataset. If the number of slices in a dataset exceeded 200, the middle 200 slices were selected, which covered the entire brain. However, if a dataset had fewer than 200 slices, an equal number of blank slices in the front and end portions of the images were introduced. Finally, because the image slices were required to be fed one by one at a time into a pre-trained VGG16 network, the images were scaled down to 224 × 224 resolution. The image preparation process began by setting the initial dimensions of each MRI image to 200 × 224 × 224. These images were then resized to improve resolution and finally cropped to a uniform 224 × 224 format.

As VGG16 input requires 3-channel 2D images, it was essential to process all gray-scale MRI image slices to make them 3-channeled before using them to train the model. Therefore, the same values were copied from the single channel onto other two channels and converted them into 3-channel images.

### 2.3. Feature Extraction

- 2D Feature Extraction with VGG16

The goal was to have an efficient process that would reduce the number of calculations and memory space requirements while producing acceptable outcomes. Therefore, an approach for extracting 2D feature maps from 3D MRI scans was used.

This technique is novel by virtue of transforming the interslice correlations of MRI images’ depth relations into correlations along a lateral direction between the successive aggregated results arising from the concatenation of 1-dimensional (1D) features obtained via assistance from VGG16 network.

To achieve this, proper preprocessing steps were applied to the MRI images and a pre-trained VGG16 model based on ImageNet was employed. To repurpose it purely as a feature extractor, the classification-relevant layers of the VGG16 model were removed and an extra layer containing 20 dense units was added to obtain extracted features. Subsequently, 20 features were extracted from each slice. In the next step, the number of slices per MRI image was changed to 200 to ensure the connection of each of these features across all the slices comprising an MRI image for creating a feature map in the form of 200 × 20 for every 3D MRI image.

**Figure 3:**
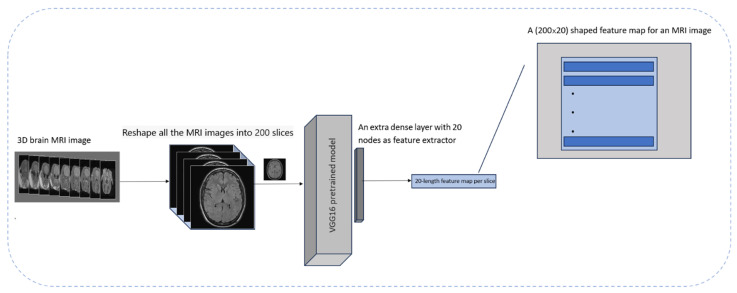
Feature extraction process of a single MRI image using VGG16 model.

To decide the number of features for each MRI slice, empirical evaluations were applied to reduce the model complexity while reducing computational time. This number of features (*n* = 20) preserves important details of the patient’s anatomy while reducing the data fitting problem. It is well documented that ADHD impacts various parts of the brain, including the prefrontal cortex, basal ganglia, and corpus callosum, which are visually identifiable in the extracted axial slices [23]. VGG16 was used in preference to other models because it was pre-trained on a large and diverse dataset, allowing for efficient feature extraction. This approach was supported by the results of the class activation mapping (CAM) technique, which confirmed that the model was focusing on the appropriate areas of the brain when making a diagnosis [24]. Moreover, this approach helps to represent features correctly and ensures calculation efficiency. This process is shown in Figure 3.

**Figure 4:**
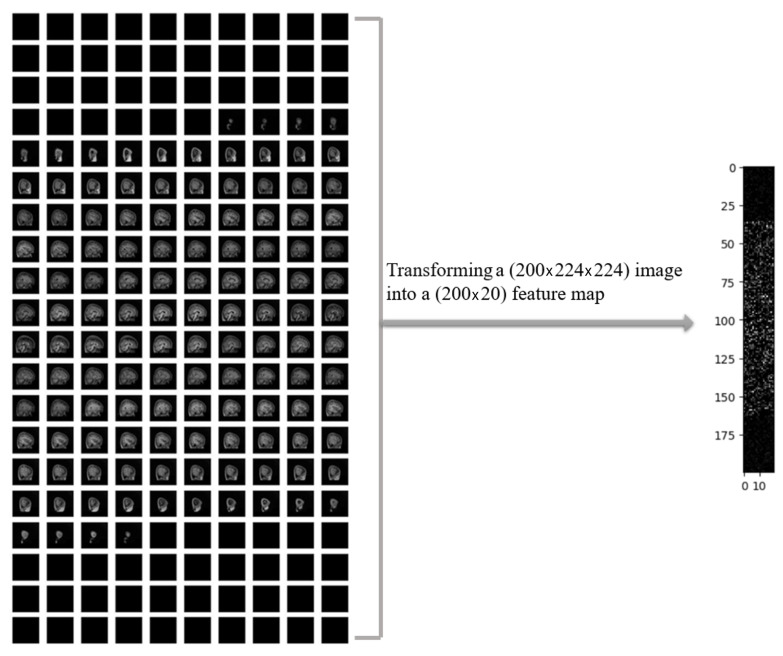
Two-dimensional extracted feature map from a single MRI with 200-slices.

In Figure 4, a sample of MRI image from the ADHD-200 dataset is presented after performing necessary preprocessing steps, resulting in 200 slices. The left panel displays the original MRI slices, while the corresponding 2D feature map is shown in the right panel. The noticeable reduction in the data size was observed by applying a one-way compression method to the original MRI images.

**Figure 5:**
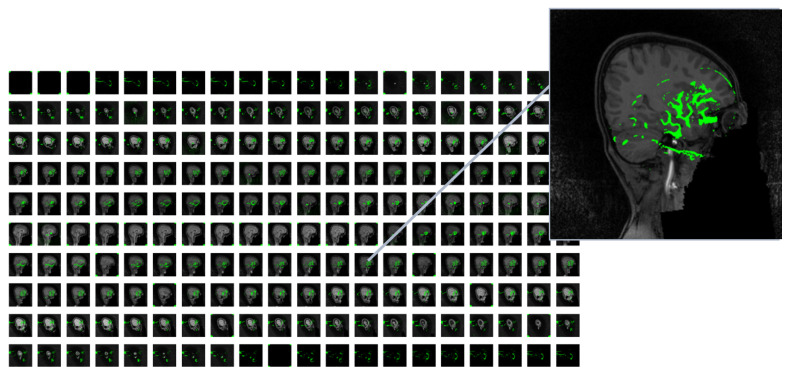
Visualization of feature activation using VGG 16-based class activation mapping (CAM) on MRI slices. The overlaid heatmap highlights regions of high significance, revealing key structures contributing to the neural network’s feature extraction.

As mentioned earlier, we extracted 20 features for each slice of every MRI image within the ADHD-200 dataset. To provide clarity regarding the regions from which these 20 features were extracted, the class activation mapping (CAM) technique was employed. The CAM offers a spatial understanding of the CNN’s decision-making process, aiding in the interpretation and explanation of model predictions [25]. CAM is a technique utilized to visualize and comprehend regions of an input image contributing the most to the final prediction of a convolutional neural network (CNN). This is achieved by obtaining an activation map through the average across all channels, indicating the importance of each spatial location. The CAM results led us to identify high-level features, including anatomical structures indicative of ADHD. As shown in Figure 5, the heatmap highlights regions in the original MRI slice that contribute the most to the activation of the last convolutional layer in the VGG16 model. The heatmap is then overlaid onto the original image. In some black slices that extend to the original 3D MRI for the purpose of standardizing sample sizes, random heatmaps may be observed. However, in slices that represent actual brain slices, we generally observe discernible areas of the brain selected for further feature extraction steps. It is important to note that this sample selection was performed at random within the entire dataset. To standardize input dimensions across subjects, black slices were added only when the original MRI volume contained fewer than 200 slices. These zero-intensity slices were symmetrically padded at the beginning and end of the volume to avoid disrupting the anatomical structure. Since these slices contain no relevant information, they are effectively treated as non-informative during training. This is further supported by CAM visualizations, which show no meaningful activation in the padded regions, confirming that the model focuses on actual brain tissue. Additionally, normalization and dropout layers help minimize the risk of these black slices interfering with feature learning.

The combination of the 2D conversion approach with pre-trained models could also be less computationally demanding compared to using 3D MRI images directly with a CNN3D network. Here are a few ways in which our approach may help reduce time complexity:

1. Three-Dimensional-to-Two-Dimensional Conversion: Converting 3D MRI scans into 2D images simplifies the data, enabling faster processing while reducing computational load.
2. Advantage of Pre-trained Models: Models like VGG16, which are already trained on previously large datasets such as ImageNet, save time and computational resources.
3. Two-Dimensional Convolutional Efficiency: Two-dimensional convolutional layers, as used in VGG16, are more computationally efficient than three-dimensional layers, accelerating the feature extraction process.
4. Parallel Processing Advantages: Many deep learning tools and hardware better support 2D data parallel processing, enhancing computational efficiency compared to more complex 3D data sets.

One of the key benefits that arises from our proposed approach is to avoid the loading and processing of whole 3D MRI volumes. Here are some key advantages of our approach in this process:

1. Computational Efficiency: Converting 3D MRI volumes to 2D feature maps significantly reduces memory usage, accelerates data loading, and simplifies model training, enabling faster and more efficient processing.
2. Scalability and Accessibility: The approach is compatible with large datasets and can be implemented on standard hardware, making it suitable for use in resource-limited clinical and research settings.
3. Simplicity and Ease of Implementation: Working with 2D data simplifies preprocessing, visualization, and debugging, making the pipeline more accessible to researchers and clinicians without specialized hardware or 3D modeling expertise.
4. Preservation of Diagnostic Information: Despite dimensionality reduction, essential anatomical features are retained, as confirmed by model performance and activation maps, ensuring reliable classification outcomes.

It is likely that this approach may make it easier to use deep learning for MRI data, especially in cases with limited computational resources.

### 2.4. Model Architecture

- Models Tested

It may be conceived that models designed to analyze spatial correlations between image pixels could detect better relationships between different regions within an image, since the objective was to investigate how well the deep connections between the slices of 3D MRI images can be translated into lateral connections in 2D feature maps. To this end, we conducted four distinct deep learning models including CNN2D, CNN1D, LSTM, and GRU. We selected CNN2D, CNN1D, LSTM, and GRU to represent a diverse range of deep learning architectures capable of capturing different structural and temporal patterns in the extracted 2D feature maps. CNN2D is well-suited for spatial pattern recognition, while CNN1D is efficient for sequential data along a single dimension. LSTM and GRU, as recurrent neural networks, are designed to model temporal dependencies and sequential relationships, which may reflect anatomical continuity across MRI slices. This diversity allows for a comprehensive comparison of spatial vs. sequential modeling strategies for ADHD classification.

**Figure 6:**
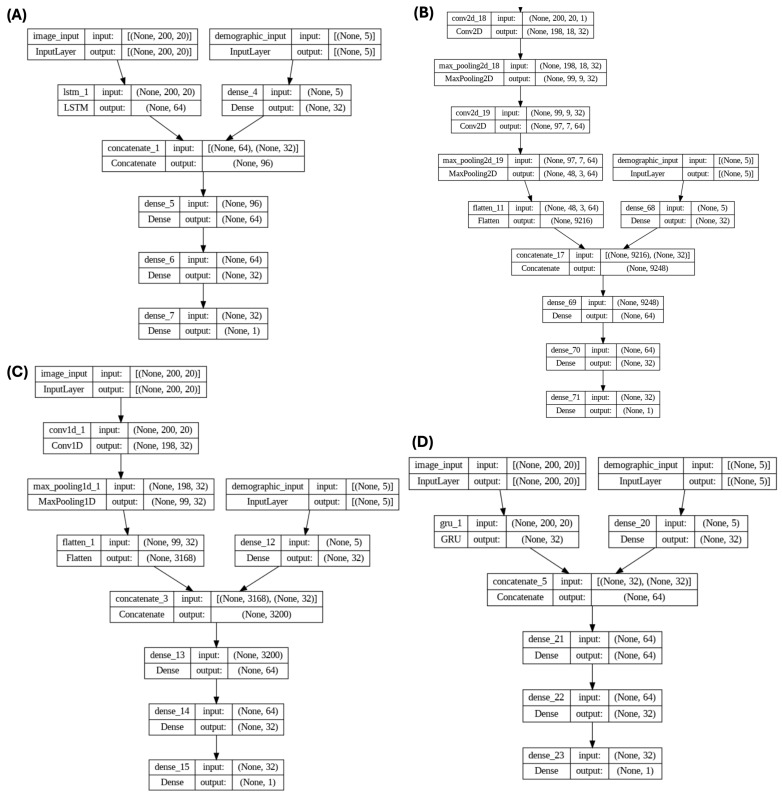
Architectural overview of the four deep learning models used in this study, each incorporating both extracted 2D feature maps from MRI images and personal attributes as input data. (**A**) LSTM model—sequential architecture designed to capture temporal dependencies across MRI slices. (**B**) CNN2D model—processes spatial patterns within 2D feature maps. (**C**) CNN1D model—sequential patterns across extracted features using 1D convolutions. (**D**) GRU model—a simplified recurrent structure capturing sequential information across slices.

These four models were investigated in two scenarios: first, considering only MRI data, and second, combining MRI data with personal attributes (reflecting the positive effects reported in the previous studies that included personal attributes along with MRI data for ADHD diagnosis [26]). The architecture of the four models, which incorporate personal attributes and extracted feature maps as input data, is depicted in Figure 6.

In this study, a total of 5 personal attributes including age, gender, full 4 IQ, handedness, and LD_or_ODD were used. It can be observed from Figure 6 that the MRI images were fed into 4 models, including GRU, LSTM, CNN2D, and CNN1D, and 2D features were extracted, followed by incorporating 5 personal attributes into the dense layers. These personal attributes along with the 2D features extracted from MRI images were used for the classification of ADHD and non-ADHD subjects.

### 2.5. Training and Evaluation

- Data Splitting

To address the issue of imbalanced datasets between the 2 labels (ADHD vs. non-ADHD), a down-sampling method was implemented to ensure an equal number of data points in each class. This process resulted in a total sample size of 172 in each class. Ten-fold cross-validation tests were applied to determine the diagnostic performance of models. Therefore, in each fold, approximately 10% of the data were randomly spilt into the testing set and the remaining 90% into the training set. In each of the 8 models (four models with and four without clinical variables), a batch size of 8 was used. Additionally, the Adam optimizer and binary cross-entropy were employed to improve training efficiency and convergence.

To reduce data overfitting, the L2 regularization technique was used as it prevents model overfitting by penalizing large weights during training. A regularization strength of 0.01 was used for all these models.

The number of epochs for the models was set to 100, but due to the use of early stopping with a patience of 10, there may be fewer epochs in each fold. In the tested models, early stopping works by tracking validation accuracy and at the end of each fold, the model weights with the best performance on this metric among the different epochs are saved.

Finally, the diagnostic performances of the models were examined by calculating metrics such as accuracy, area under the ROC curve (AUC), F1-score (the harmonic mean of precision (positive predictive value), and recall (sensitivity), and the results of each fold were also compared for a more precise and general evaluation.

- Loss function

We used the binary cross-entropy loss function, which is standard for binary classification tasks like ADHD vs. non-ADHD. This loss function measures the difference between the predicted probability and the actual label, penalizing incorrect predictions more heavily as confidence increases. It was chosen for its effectiveness in optimizing probabilistic outputs in deep learning models and its compatibility with sigmoid-activated output layers used in our classification networks.

## 3. Results

**Figure 7:**
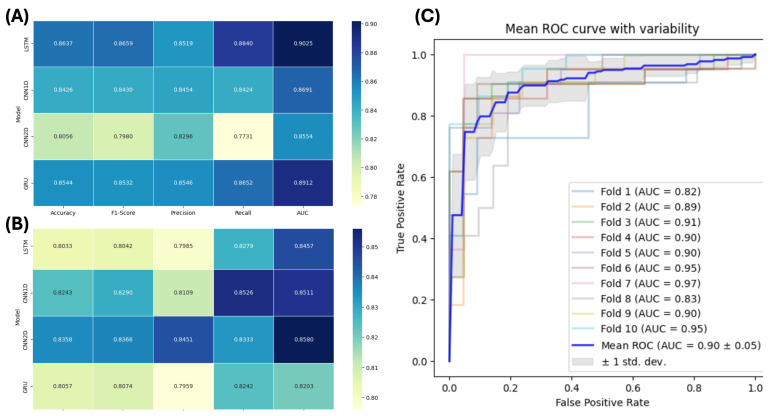
(**A**) Average results heatmap using 10-fold CV (input data: 2D images + demographic featurs). (**B**) Average results heatmap using 10-fold CV (input data: 2D images). (**C**) Mean area under the ROC curve (AUC) plot for each fold of cross-validation of LSTM model (the best model observed in our study).

Figure 7A depicts the heatmap of the average results of the 10-fold cross-validation using 2D images derived from 3D MRI images with clinical variables. Based on our results, the LSTM model had the best overall diagnostic performance. This model provided the highest scores across multiple metrics—accuracy, F1-Score, recall, and AUC—indicating superior performance compared to other models. The GRU model was found to be the second-best performing model.

Figure 7B shows the heatmap of the average results of the 10-fold cross-validation using only 2D images derived from 3D MRI image without including clinical variables. The CNN2D model showed the highest diagnostic performance. This model provided high accuracy, F1-Score, and AUC. The CNN1D model was the second-best performing model in a group of models, achieving the second-highest accuracy and AUC and the highest recall among all models tested.

Figure 7C presents the mean AUC plot for each fold of the 10-fold cross-validation test conducted on the LSTM model. Figure 7C demonstrates 5% variations in performance between different folds, suggesting that the model was not overfitting to a specific subset of the data and generalized well across different splits of the data.

## 4. Discussion

This study focused on the improvement of ADHD diagnosis by incorporating modern deep learning-based methods utilizing MRI images with or without clinical data. ADHD diagnosis can be complex due to its various subtypes and the subjective nature of the diagnostic process that is observer- or evaluator-dependent. Furthermore, neuroimaging techniques such as brain MRI can be constrained by reliable visual inspection of subtle anatomical details, potentially impacting the accurate diagnosis. However, the use of AI and machine/deep learning-based methods can improve patient diagnosis and clinical decision-making by identifying specific imaging biomarkers or signature patterns.

In a deep learning resting-state functional MRI (rsfMRI) study called “DeepFMRI”, using the same dataset (ADHD-200), Riaz et al. [27] reported that the deep learning method based on functional connectivity was able to achieve a classification accuracy of only 73.1% (sensitivity 65.5% and specificity 91.6%). In another study, investigators [28] applied a support vector machine (SVM) classifier and an extreme learning machine to cortical MRI data from 159 children in the ADHD-200 dataset, which included typically developing (TDC), ADHD-inattentive (ADHD-I), and ADHD-combined (ADHD-C) types. Using SVM-based recursive feature elimination (RFE-SVM) for feature selection, the approach achieved a moderate classification accuracy of 0.61. For differentiating ADHD patients from healthy controls, Chen et al. [29] employed rsFMRI and a dual subspace classification algorithm. This method provided a classification rate of 0.90 on the ADHD-200 dataset using a binary hypothesis test to determine the projected energy of FCs in two different spaces being representative of ADHD and control. Dey et al. [30] presented a novel framework for classifying ADHD subjects using rsfMRI data by constructing brain functional connectivity networks and applying a Support Vector Machine (SVM) classifier on low-dimensional projections via multi-dimensional scaling (MDS). The approach achieved classification accuracies of 0.70 on the training set and 0.74 on the testing set, with higher detection rates when male and female subjects were classified separately. Unlike the study by Choi et al. [13], which used pupil size dynamics from eye-tracking data, our manuscript focuses on structural MRI, with significantly larger and more diverse data (758 subjects vs. 50). While their model achieves an AUC of 0.86 on task-based physiological data, our LSTM model achieved an AUC of 0.90 using anatomical features and clinical variables. Our method emphasizes clinical scalability through reduced computational load and integration with standard neuroimaging workflows.

Our study provides certain important refinements to prior studies, aiming to improve the diagnosis of ADHD using AI and neuroimaging data. In comparison to our findings, previous studies achieved lower classification accuracies and AUCs. A prior work provided a diagnosis AUC of 0.80 and classification accuracy of 0.75 for ADHD diagnosis, mainly using rsfMRI data. On the contrary, our approach using clinical variables and anatomical MRI with the LSTM network provided better AUC (0.90) and accuracy (0.86). In addition, with only structural and spatial patterns embedded in the MRI data, the CNN2D model provided an accuracy of 0.84 and an AUC of 0.86. These results are encouraging as they outperformed previous benchmarks even without the inclusion of clinical or demographic information.

The improved performances of models in our study highlight the effectiveness of combining clinical information and deep neural networks-based image analysis. Such an integrative approach offers a good foundation for future development of reliable, generic, and widely applicable ADHD diagnostic approaches. Given that data were collected from various international research centers using different scanner vendors and acquisition parameters, as well as from different patient population, all these factors significantly enhance the reliability and generalizability of our proposed methodology. However, future clinical trials or studies are required to validate our promising findings. The close range of AUC in Figure 7A also shows the capability of our model to generalize well, even without incorporation of clinical data, emphasizing the fact that this approach can be applied across diverse clinical settings. The preprocessing strategies are of great importance in model performance. We were successful in reducing the computational costs by using VGG16 as a feature extractor. Using 2D convolutional layers instead of 3D MRI scan conversion to 2D feature maps significantly increased computational performance and provided support for parallel processing of data that are easier to manage in 2D format. While the dimensionality was reduced by converting 3D MRI volumes into 2D feature maps, it was ensured that the critical anatomical information remains preserved, which was required for accurate analysis. The selection of 200 central slices per subject retained the most representative brain structures, and feature extraction via a pre-trained VGG16 network focused on regions known to be associated with ADHD, such as the prefrontal cortex and corpus callosum. This was further supported by CAM, which demonstrated that the extracted features consistently highlighted diagnostically relevant regions, confirming minimal spatial feature loss.

The improved diagnostic performance of the LSTM model may be attributed to several methodological refinements used in this study. Most notably, the inclusion of demographic variables in our LSTM model may seem to have extracted other related contextual information, enriching the model’s learning ability for associating with ADHD symptoms, leading to better diagnostic accuracy. This is consistent with findings in the literature, which demonstrate that demographic factors like age, gender, IQ, and handedness are closely linked with ADHD. However, the GRU model performed poorly across various metrics when personal attributes were not considered. These key observations suggest that LSTM and GRU’s inherent architectures were made more effective by the additional information provided by demographic variables. While VGG16 has higher training costs, our pipeline uses it only as a fixed feature extractor (not for training), which significantly reduces training time compared to full CNN training from scratch. The overall pipeline is optimized for clinical scalability.

In summary, the inclusion of demographic attributes in deep learning systems, especially LSTM and GRU architectures, appears promising for the detection of ADHD via MRI analysis. Nevertheless, the CNN models showed an ability to compensate for missing elements and, in some cases, even outperformed other models, emphasizing the importance of model selection based on the properties of the data being analyzed. This investigation provides a basis for future studies to investigate AI-driven, non-intrusive diagnostic tools for ADHD that will be more accurate, efficient, and accessible than current approaches. The proposed framework is designed with practical implementation in mind. By transforming 3D MRI data into compact 2D feature maps and leveraging pre-trained models, our approach significantly reduces computational requirements, making it feasible for integration into existing radiology software or clinical decision-support systems. This streamlined pipeline enables more accessible, faster, and scalable ADHD diagnosis in real-world clinical settings, particularly in environments with limited computational resources.

Future studies can build upon current findings in various directions, including distinction between various subtypes of ADHD like inattentive, hyperactive–impulsive, or combined types. This extra detail would enhance the practical utility of models in clinical settings and enable further in-depth understanding of various manifestations of ADHD. While the ADHD200 dataset is not recent, it is still a valuable source as it provides a large collection of neuroimaging data from ADHD patients and unaffected controls. Due to a limited sample size and down-sampling, we could not compute reliable confidence intervals. Future studies with larger datasets are needed to validate the statistical robustness.

## 5. Conclusions

This study provides an important step towards improving ADHD diagnosis by applying modern deep learning techniques to MRI data and clinical characteristics. Our best-performing model (LSTM with clinical features) achieved an accuracy of 0.86 and an AUC of 0.90, outperforming previously reported benchmarks. The integration of demographic variables significantly enhanced the model performance and offers a promising path to non-invasive, AI-based diagnosis of ADHD. However, this study is limited by the use of a single dataset and the lack of external validation. Future studies should test the diagnostic performance of our model across independent datasets to establish its broader generalizability and wider clinical adoption.

## Data Availability

The data analyzed in this study are publicly available as part of the ADHD-200 Global Competition dataset at http://fcon_1000.projects.nitrc.org/indi/adhd200/ (accessed on 11 October 2023).
